# Biodegradation and hydrolysis of rice straw with corn steep liquor and urea-alkali pretreatment

**DOI:** 10.3389/fnut.2022.989239

**Published:** 2022-08-04

**Authors:** Yulin Ma, Xu Chen, Muhammad Zahoor Khan, Jianxin Xiao, Shuai Liu, Jingjun Wang, Gibson Maswayi Alugongo, Zhijun Cao

**Affiliations:** ^1^State Key Laboratory of Animal Nutrition, College of Animal Science and Technology, China Agricultural University, Beijing, China; ^2^Department of Animal Breeding and Genetics, Faculty of Veterinary and Animal Sciences, University of Agriculture, Dera Ismail Khan, Pakistan

**Keywords:** rice straw, corn steep liquor, ruminal bacteria, CSL and urea-alkali pretreatment, enzymatic hydrolysis

## Abstract

The current study evaluated the corn steep liquor (CSL) and urea-alkali pretreatment effect to enhance biodegradation and hydrolysis of rice straw (RS) by ruminal microbiome. The first used RS (1) without (Con) or with additives of (2) 4% CaO (Ca), (3) 2.5% urea plus 4% CaO (UCa) and (4) 9% corn steep liquor + 2.5% urea + 4% CaO (CUCa), and then the efficacy of CSL plus urea-alkali pretreatment was evaluated both *in vitro* and *in vivo*. The Scanning electron microscopy, X-ray diffraction analysis, cellulose degree of polymerization and Fourier-transform infrared spectroscopy, respectively, results showed that Ca, UCa, and CUCa pretreatment altered the physical and chemical structure of RS. CSL plus Urea-alkali pretreated enhanced microbial colonization by improving the enzymolysis efficiency of RS, and specially induced adhesion of *Carnobacterium* and *Staphylococcus*. The CUCa pretreatment could be developed to improve RS nutritional value as forage for ruminants, or as feedstock for biofuel production.

## Graphical Abstract

Biodegradation and hydrolysis of rice straw with corn steep liquor and urea-alkali pretreatment. 
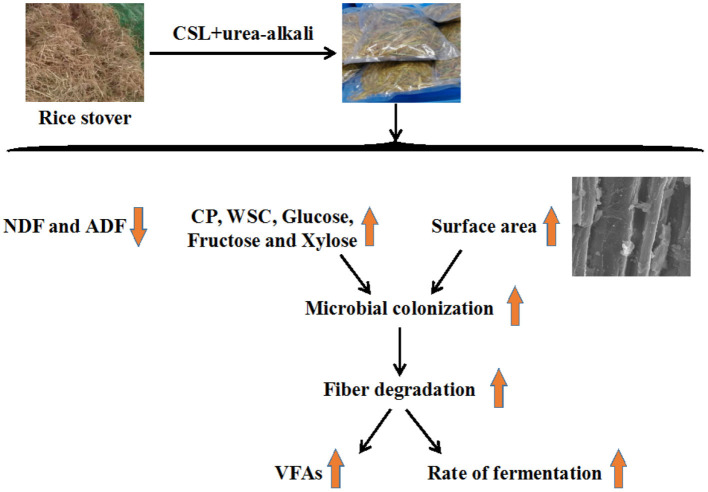

## Introduction

Rice is one of the main food crops, and about 115 million hectares of the earth is covered by paddy fields (1). Currently, more than 30% of rice straw in China is either burned or left on the field, which is not only contribute to waste of biomass resource but also decreasing the air quality and affecting health of human (2, 3). The efficient utilization of rice straw for livestock production and biofuel could combat the energy crisis and feed shortage and consequently reduces the environmental pollution (4). At present, 20% of annual rice straw production in China is under use as animal feed, which is only enough for half of animal roughage requirements. Straw resources have the potential to increase ruminant production in China, but efficient strategies are needed to improve the utilization and nutritional values of rice straw. Rice straw is high in carbohydrates such as cellulose and hemicellulose, which can be used as a better energy source for ruminants. However, lignin encapsulates cellulose and hemicellulose to form a polymer, which prevents rumen microorganisms from entering into the rice straw to perform their degradation function. Therefore, rice straw shows lower nutrient utilization and feed value (5, 6). Previous studies have exercised various pretreatment approaches including physical, chemical, and biological methods to improve the digestibility of rice straw by reducing lignin (7, 8).

Chemical pretreatment is one of the most widely used methods, which could improve the digestibility of rice straw. However, limited information is available on the improvement of alkali or urea alone treatment on digestibility and nutritional value of rice straw (9, 10). It has been reported that the combined pretreatment of urea and alkali could improve nutritive value of rice straw, because alkali can destroy lignocellulose structure, and urea can increase the crude protein content of rice straw. Furthermore, combined pretreatment of urea and alkali leads to the improvement of the nutritional value of rice straw (11) by degrading lignin and promoting the release of cellulose and hemicellulose from lignocellulose used by rumen microbes and promote the transformation of biological energy (12). However, only 30–35% of NH_3_ is retained during urea pretreatment (13), the rest is released into the atmosphere as amines, which may contribute to pollute air quality and loss of nitrogen resources. Meanwhile, feeding urea pretreated rice straw to the cows, would lead to rapid liberation of the retained nitrogen in the rumen and cause nutrient losses (14).

Corn steep liquor (CSL) is an activated sludge, which is from agriculture, dairy and bread industries and can be used as high nitrogen nutrients source (15). In addition, CSL is acidic and has the function of fixing nitrogen, and can retard nitrogen liberation in the rumen, enhance the absorption of feed and improve the health of animals. If utilized properly, these by-products can protect the environment through waste recycling. In addition, CSL from wet milling by-products of the corn industry can provide rich nutritional supplements to promote fermentation and microbial growth (16). Notably, CSL has high amount of trace and mineral elements as a source of organic nitrogen, which is used as an inexpensive nutrient for the microbial production of enzymes (17). It is worth noting that in the future, CSL could consider for improving the degradation of lignocellulose resources by rumen microorganisms and enhancing the efficiency of bioenergy conversion.

The microbiota in the rumen degrades cellulose mainly by colonizing the surface of ingested forages and secreting fiber-degrading enzymes (18). The rumen bacterial communities' members vary in their preferences for feed particles and rumen wall attachment (19–21). The rumen microbial communities' attachment to feed particles is an essential step in the rumen fermentation and digestion processes (22). The community composition of particle attached to microbes also differs among feeds and is likely effected by the feed chemical compositions (23). However, there is limited information on the effect of pretreated rice straw on the colonization of microbes in the rumen, and most studies focused on the raw feed materials (20). In addition, the chemical characteristics and physical structure of the feed particles are main parameters that effect the microbial colonization (24). Therefore, further evaluation of bacterial attachment changes after pretreatment is of great significance for improving the nutrient utilization efficiency of ruminant feed. Indeed, volatile fatty acids (VFAs) have been widely used for bioenergy production as a product of rumen microbial degradation of feed (25–27). In the present study, the CSL and urea-alkali pretreated rice straw with respect to its chemical composition, microbial digestibility (*in vitro*), enzymatic hydrolysis, physical and chemical structure, and microbial colonization (*in vivo*) were characterized.

## Materials and methods

### Ethical statement

The Animal Care Committee of the School of Animal Science and Technology, China Agricultural University (Protocol Number: 2013-5-LZ) has reviewed and approved all the animals used in this study.

### Raw material preparation

Rice straw was randomly collected from suburban farm Land (32.13°N, 114.07°E Gushi County, Xinyang City, Henan Province, China) on December 10, 2019. The samples were stored at room temperature in a sealed plastic bags a before use. The CSL, urea, and CaO were provided by Henan Yuyao New Medicine Co. LTD and Henan Hand-in-Hand Fertilizer Co. LTD (Henan, China), respectively.

### Anaerobic CaO and urea alkalization treatment

A total of 500 g rice straw was weighted and chopped into 2–3 cm lengths, and stored in laboratory polythylenea 25 × 35 cm sterile bags provided by Beijing Shengya Yuda Biological Technology Co., Ltd. (Beijing, China); a total of 180 bags of rice straw were prepared. These bags were pretreated with four different approaches based on dry matter (DM): (i) no pretreatment for control group (Con); (ii) 4% CaO (Ca); (iii) 2.5% urea and 4% CaO (UCa); and (iv) 9% CSL+ 2.5% urea + 4% CaO (CUCa). The urea, CaO, CSL, and water quantity were calculated in advance and weighed for each treatment. Then, the additives were sprayed evenly on the surface of the rice straws and mixed well. Distilled water was added to the material to adjust the moisture to 45%, then stored in polyethylene sterile bags and sealed with a food vacuum sealer (Konka KZ-ZK007; Dongguan Yijian Packaging Machinery Co. Ltd., Dongguan, China) and stored at ambient temperature (25 ± 3°C) for 15 days. Each approach was contained of 15 bags. Subsequently, part of the samples were dried (65°C, 48 h) and ground in a hammer mill, passed through a 1 mm sieve, and then analyzed for chemical composition, structural changes and enzymatic hydrolysis. In addition, another part of the samples was pretreated by passing a 2.5 mm sieve before evaluating *in vitro* digestibility and microbial communities attached to the rice straw. Finally, each sample was tested in triplicate.

### Analysis of physical and chemical composition changes of CSL plus urea-alkali pretreated rice straw

The crude protein (CP), crude ash content (Ash) and DM, of the rice straw samples were measured through the method adopted by AOAC (28). The content of neutral detergent fiber (NDF) and acid detergent fiber (ADF) was analyzed by methods established in previous studies (29) and detected using an Ankom 2000i Fiber Analyzer (Ankom Technologies, Macedon, NY, USA). Alteration in the physical structure of straw before and after pretreatment was analyzed by scanning electron microscopy (SEM). Briefly, untreated rice straw samples and Ca, UCa, CUCa-treated rice straw samples were set for 4 h in 1% osmium tetroxide solution and treated with different concentrations of ethanol (20, 40, 60, 80, and 100%) were dehydrated and washed with phosphate-buffered saline for 10 min (3 times in 100% ethanol). The dehydrated samples were carried out in carbon dioxide using an AutoSamdri®-815 critical point dryer (Tousimis, Rockville, USA). Stick double-sided conductive adhesive on the sample stage, and stick the surface of the sample to be observed up on the conductive adhesive. When sticking the sample, do not touch the surface of the sample to be observed. To observe the cross-section of the sample, slide the cross-section of the sample with a double-sided blade and stick the sample sheet on the conductive adhesive with the cross-section up. Each dried sample was sputtered with gold and palladium using a 208HR sputter coater (Cressington, Waterford, UK). The sample stage is fixed on the sample holder, put into the SEM sample chamber, and then evacuated and high pressure treated. Samples were observed for morphological changes at 1500× magnification.

The polymerization (DP) degree of rice straw samples was measured through viscosity method (30) with minor modifications described (31). The method is based on the relationship: DP^0.905^ = 0. 75 [η], and the [η] is the intrinsic viscosity of the solution. Briefly, all processes were conducted at 25 ± 0.5°C, with the Ubbelohde viscosity meter and cupriethylenediamine hydroxide (Cuen) as solvent. The intrinsic viscosity was calculated by interpolation using the USP table (32), showing the predetermined values of the product of intrinsic viscosity and concentration. The [η] C, for cellulose samples exhibiting relative viscosity (η_rel_) values between 1.1 and 9.9. η_rel_ was calculated using the equation: η_rel_ = t/t_0_, where t and t_0_ are the efflux times for the cellulose solution and Cuen (blank) solvent, respectively. All experiments were performed in technological triplicate.

The cellulose crystallinity index (CrI) of rice straw, after and before CSL plus urea-alkali pretreatment was measured by Siemens D-5000 diffractometer (Bruker, Ettlingen, Germany). The rice straw samples were scanned from 3° to 40° with a step size of 0.02 and 3 s per step at a voltage of 40 kV and 20 mA. The CrI was calculated as described previously (33).

The chemical structure and composition of the untreated rice straw and the CSL and urea-alkali pretreated were analyzed using FTIR spectrophotometer (Bruker, Ettlingen, Germany) equipped with an RT-DLaTGS detector at 4,000–1,000 cm^−1^ with a resolution of 4 cm^−1^ and 16 scans per sample. Fine ground samples (200 meshes; 1.0 mg) were mixed with KBr (50 mg) before scanning, and pressed into a pellet for analysis at the 1 MPa of pressure.

### *In vitro* digestibility and gas production performance

#### Rumen fluids collection

Three Holstein cows weighing ~650 ± 45 kg/cow, lactation stage, day of lactation was 152 ± 14 and milk yield of 38 ± 3 kg/day, equipped with permanent rumen fistulas were used as rumen fluid donor animals. Cows were fed three times a day (07:30, 14:30, and 18:30) with free access to water. The basic diet and nutrient levels have been provided in Supplementary Table S1.

#### *In vitro* rumen incubation

In the current study, the AGRS-(Automated Trace Gas Recording System) type microbial fermentation gas production system was used to detect the cumulative gas production (GP) (34). Rumen fluids from three Holstein cows were collected 2 h after feeding in the morning, placed at 39°C in a pre-temperature vacuum flask, and immediately transferred to the laboratory. The rumen fluids of the three cows were mixed in equal proportions, squeezed through four layers of mussels before *in vitro* inoculation and placed in a CO_2_ bath at 39°C. Each bottle (120 mL; 6 replicates/samples) was filled with 0.5 g of sample, 25 mL stratified rumen fluid and 50 mL medium (pH 6.85). The medium was prepared according to the method developed by Menke and Steingass (35). All bottles were purged with anaerobic N_2_ for 5 s, sealed with rubber plug and Hungate screw caps and individually connected with medical plastic infusion pipes to the AGRS, using the previously adopted method (36). All the bottles were incubated at 39°C for 48 h, and each batch culture system runs four bottles of rice straw samples for blank correction.

After incubation for 48 h, the bottle was removed from the AGRS system. The pH value of the culture medium was immediately measured using S400-B [Mettler Toledo Technology (China) Co., LTD]; The cultured medium (1 mL) was mixed with 0.3 mL interphosphate solution containing 2.5 g/L at 4°C for 30 min, and centrifuged at 10,000 × g at 4°C for 10 min. The supernatant was stored at −20°C for the measurement of acetate acid (AA), propionic acid (PA), butyrate acid (BA) and total volatile fatty acids (TVFA). Remove the supernatant from the bottle and dry the remaining residue at 65°C to constant weight for DM and NDF measurements. *In vitro* disappearance of DM (IVDMD), NDF (IVNDFD), and ADF (IVADFD) was calculated as a difference between the initial culture of DM, NDF and ADF, residual DM, NDF, and ADF, corrected by blanks.

#### Computation

Cumulative gas production has been recorded using the AGRS-III microbial fermentation gas production system and obtained according to the exponential function model proposed by Jcj et al. (37);

(1) $$\begin{array}{r} GP_{\text{t}}=GP_{\text{max}}/\left[1+\;{\left(C/t\right)}^{\text{B}}\right] \end{array}$$

The GP_t_ is the total gas production (mL/g DM) at time t; GP_max_ is the theoretical maximum gas production (mL/g DM) at a constant fractional rate (c) per unit time; “t” is the gas recording time, and B represents a sharpness parameter determining the shape of the curve, and C is the time (h) at which half of GP_max_ is reached.

### Enzymatic hydrolysis

Enzymatic hydrolysis was carried out through a commercial β-glucosidase preparation and cellulolytic enzyme mixture SAE0020 (Sigma) as proposed by previous study (7). A solid loading of 5% (w/v) and enzyme loadings of 20 FPU/g and 15 CBU/g dry matter (DM) in 125 mL Erlenmeyer flasks were used for enzymatic hydrolysis. Furthermore, the reaction was carried out in 50 mM sodium citrate buffer at pH 4.8 and incubated in a thermostated air bath shaker setting at 50°C and 180 rpm/min for 72 h. To stop the possible contamination due to microbes, contamination, 0.02% (v/v) ProClin was added to the hydrolysate before adding the enzyme. The enzyme blank without substrate was conducted in parallel with other samples. Samples (1 mL) were taken with the pipette to cut the tip at 72 h of incubation. Subsequently, the enzymatic hydrolysate was centrifuged at 3,000 × g for 5 min, and WSC yield was determined with the supernatant and expressed on a dry matter basis (mg/g DM). Briefly, take 0.5 mL of sample solution, add 1 mL of water and 1.5 mL of DNS solution to the colorimetric tube, shake well, heat in a boiling water bath for 5 min, cool with cold water for about 10 min to room temperature, add 22 mL of water to dilute to 25 mL. Add 1.5 mL of DNS solution to 23.5 mL of water as blank, and measure the absorbance at a wavelength of 540 nm. The measured linear regression equation of absorbance (y) and glucose content (x) is

y = 0.2004x – 0.0008

The linear range of this equation is 0–1.6, and the correlation coefficient R = 0.999247.

The DNS solution preparation process is to weigh 6.3 g of 3, 5-dinitrosalicylic acid, 182 g of potassium sodium tartrate, 5 g of redistilled phenol, 21 g of sodium hydroxide, and 5 g of sodium hydrogen sulfite, mix and heat to dissolve and set the volume to 1,000 mL. Store in a brown bottle for later use.

### *In situ* rumen incubation and sample collection of the rice straw

The rice straw was dried at 65°C, ground into ~0.5 mm pieces, and weighed into individual nylon bags (0.5 g/bag). The samples of four groups were put into nylon bags, and each group was set with 2 duplicate bags, which were randomly put into the rumen of 3 Holstein cows with permanent fistula, and incubated for 0.5, 4, 12, and 24 h respectively. The cows' basic diet composition and nutrient value showed Supplementary Table S1.

The determination of rumen microorganisms colonized on the surface of rice straw samples was based on the method Liu et al. (20) and slightly modified. Briefly, two nylon bags from each of the four pretreatment rice straw were retrieved from each rumen after 0.5, 4, 12, and 24 h of the incubation and washed three times with phosphate-buffered saline (PBS, pH 7.4) to remove liquid-borne and loosely attached microbes and finally squeezed by hand with sterile gloves to remove excess water. The samples were then transferred in liquid nitrogen to the laboratory and were stored at −80°C for subsequent DNA extraction.

### Microbial diversity analysis

For analyzing the microbial community, the EZNA stool DNA Kit (Omega Biotek, Norcross, GA, U.S.) was used to extract microbial DNA. For bacteria, the V3-V4 variable region of the 16S rDNA was targeted using primers Eub338F (ACTCCTACGGGAGGCAGCAG) and Eub806R (GGACTACHVGGGTWTCTAAT), which generated a fragment of 460 base pairs (bp) suitable for paired-end sequencing the Illumina Miseq system (Shanghai Majorbio Bio-pharm Technology Co., Ltd). The PCR condition consisted of an initial denaturation at 95°C for 5 min followed by 35 cycles at 95°C for 30 s, at 58°C for 30 s and 72°C for 1 min, and a final extension at 72°C for 5 min. The reactions were performed in a 20 μL mixture containing 10 μL of 2X Taq Plus Master Mix, 0.8 μL of each primer (5 uM), 7.4 μL of ddH_2_O, and 1 μL of each reaction was used as a template of PCR. Each sample was performed in triplicate of PCR reactions.

### Sequence analysis

The raw 16S rRNA gene sequencing reads were demultiplexed, quality-filtered by fastp version 0.20.0 (38) and merged by FLASH version 1.2.7 (39) with the following criteria: (i) the 300 bp reads were truncated at any site receiving an average quality score of <20 over a 50 bp sliding window, and the truncated reads shorter than 50 bp were discarded, reads containing ambiguous characters were also discarded; (ii) only overlapping sequences longer than 10 bp were assembled according to their overlapped sequence. The maximum mismatch ratio of the overlap region is 0.2. Reads that could not be assembled were discarded; (iii) Samples were distinguished according to the barcode and primers, and the sequence direction was adjusted, exact barcode matching, and two nucleotide mismatches in primer matching.

Operational taxonomic units (OTUs) with a 97% similarity cutoff (40) were clustered using UPARSE version 7.1 (41), and chimeric sequences were identified and removed. The taxonomy of each OTU representative sequence was analyzed by RDP Classifier version 2.2 (42) against the 16S rRNA database (e.g., Silva v138) using a confidence threshold of 0.7.

### Statistical analysis

All the data were analyzed using the IBM SPSS Statistics 24 (SPSS Inc., Chicago, IL, USA). One-way ANOVA analysis was performed to examine the effect of CSL plus urea-alkali pretreatment on the chemical composition, physicochemical structure, enzymatic hydrolysis, and *in vitro* digestibility of rice straw of rice straw. In addition, the Duncan multiple comparison method was carried out to compare the differences between the means; *P* < 0.05 was used to show significance levels.

## Results and discussion

### Change in the chemical composition of rice straw after CSL plus urea-alkali pretreatment

The chemical composition of the rice straw both before and after the CSL plus urea-alkali pretreatment has been summarized in Table 1. The NDF and ADF contents were significantly (*P* < 0.05) decreased by the Ca, UCa, and CUCa pretreatment, 70.16–63.79%, and 44.29–39.21% for NDF and ADF, respectively. And the CP content was increased (*P* < 0.001) from 5.45 to 8.00% in CUCa. These findings showed that the NDF and ADF contents of rice straw were hydrolyzed and/or removed to a greater extent in combined treatment of CSL plus urea-alkali than alone; while the ability of nitrogen fixation was remarkable. Constantly, a study reported that the NDF and ADF contents were reduced by alkaline pretreatment by dissolving a part of cellulose and hemicellulose (43).

**Table 1:** Effect of CSL plus urea-alkali pretreatment on the chemical composition of rice straw.

**Items**	**Treatment**				**SEM**	***P*-value**
	**Con**	**Ca**	**UCa**	**CUCa**		
DM, %	97.35^a^	97.15^ab^	96.67^c^	96.84^bc^	0.071	0.015
CP, %DM	5.45^c^	5.39^c^	7.22^b^	8.00^a^	0.112	<0.001
NDF, %DM	70.16^a^	65.32^b^	64.80^b^	63.79^b^	0.613	0.012
ADF, %DM	44.29^a^	40.98^b^	40.25^b^	39.21^b^	0.341	0.002
EE, %DM	14.51	16.52	15.61	14.84	0.142	0.577
Ash, %DM	2.45^c^	2.14^a^	2.42^b^	2.47^bc^	0.112	0.002

Different superscript letters a, b, and c indicate significantly different values (P < 0.05) across-rows, and the same or no letters indicate insignificant differences (P > 0.05).

Con: no pretreatment for control group, Ca: 4% CaO, UCa: 2.5%urea + 4% CaO, CUCa: 9% CSL + 2.5% urea + 4% CaO.

DM, dry matter (the dry matter content is calculated based on air-drying the sample); CP, crude protein; NDF, neutral detergent fiber; ADF, acid detergent fiber; The CP, Ash, NDF and ADF content are all calculated based on absolute dry matter. SEM, standard error of means.

### Association of CSL plus urea-alkali pretreatment with structural changes of rice straw

#### Changes in chemical structures

**Figure 1:**
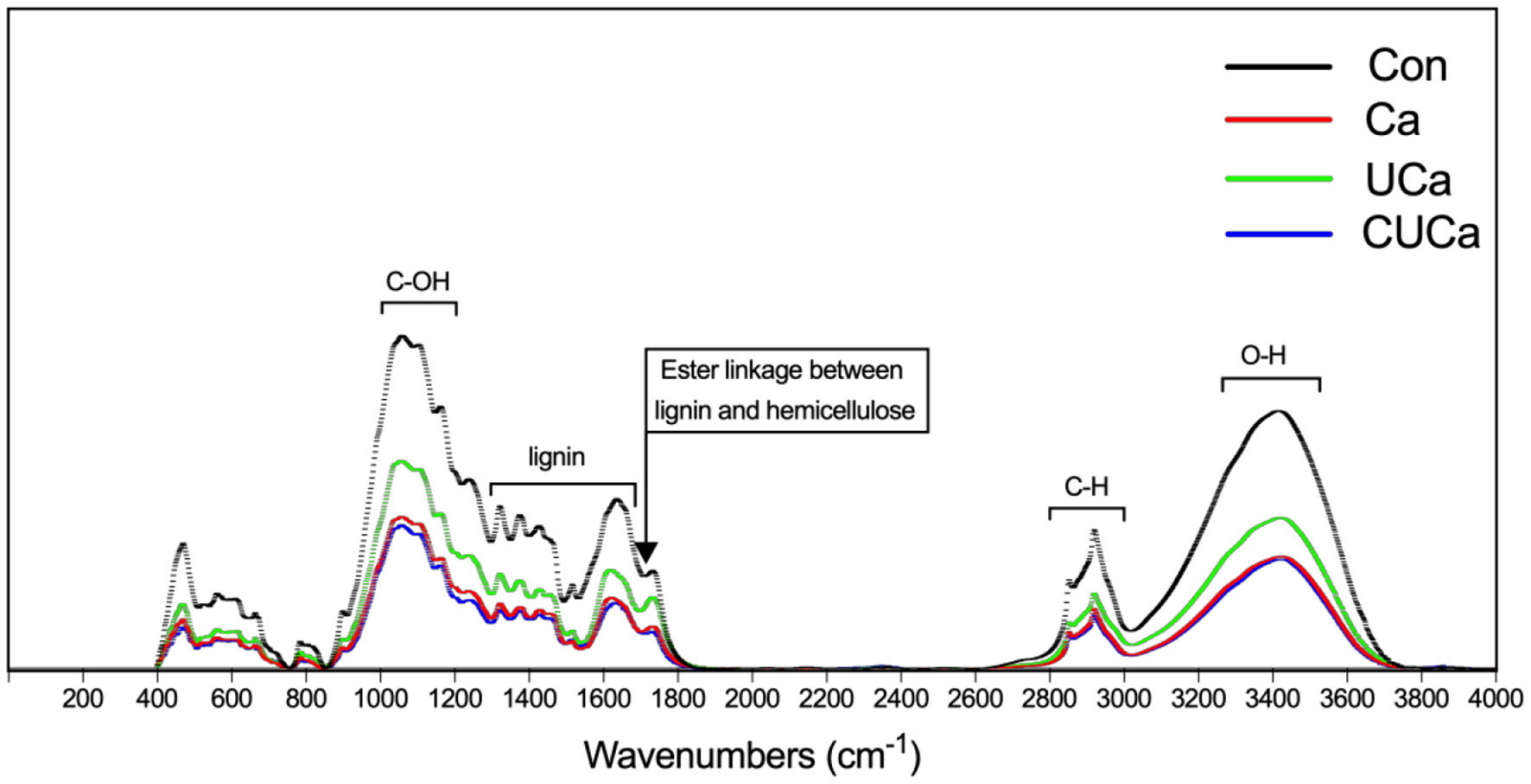
FTIR spectroscopy of rice straw after CSL plus urea-alkali pretreated. Con: no pretreatment for control group, Ca: 4% CaO, UCa: 2.5% urea + 4% CaO, CUCa: 9% CSL + 2.5% urea + 4% CaO.

FTIR spectroscopy was used to examine modifications to the chemically functional groups of rice straw following CSL plus urea-alkali pretreatment (Figure 1). According to Rosa et al. (44), the broad band at 3,350 cm^−1^ was related to the O-H stretching of the hydrogen bonds in cellulose, hemicellulose, and lignin, which decreased following Ca, UCa, and CUCa pretreatments because hemicellulose and lignin from rice straw were partially removed. The band at 2,900 cm^−1^ was attributed to C-H stretching within the wax, and the decrease after pretreatment was due to the removal of wax from rice straw (45). Furthermore, the band at 1,200–1,000 cm^−1^ was typically related to the C-O-H stretching of cellulose and hemicelluloses. The vibrations of these bands overlapped the aromatic C-H in-plane deformation from guaiacyl type lignin at 1,040 cm^−1^, C-O-C glycosidic linkage at 1,160 cm^−1^ and C-O-C ring skeletal vibration at 1,100 cm^−1^ (46), and the decrease in bands after pretreatment indicated the partial removal of lignin and hemicellulose from rice straw. This is supported by the fact that the adsorption peaks of bands at 1,740–1,245 cm^−1^, which corresponded to the stretching of the acetyl group in hemicellulose and aromatic ring vibration of guaiacyl lignin, respectively. In addition, the band at 1,720 cm^−1^ was usually defined C-O of the ester linkages the acetyl group in hemicelluloses structure and/or the linkage between lignin and hemicelluloses (47). In our study the decrease in the band after pretreatment suggests the occurrence of the absorption peak at 1,720 cm^−1^ may reveal the presence of remaining ester linkage between lignin and hemicelluloses. Furthermore, we analyzed the distribution of lignin-related bands. The bands were defined as aromatic skeletal stretching at 1,610 and 1,516 cm^−1^ (48). Meanwhile, the bands at 2,860, 1,460, and 1,425 cm^−1^ have been related to C-H deformation within the methoxyl of lignin (49). Since the adsorption of these bands as mentioned above were decreased after pretreatment, it is suggested that CSL plus urea-alkali pretreatment could remove lignin from rice straw. Furthermore, the band at 1,640 cm^−1^ was attributed to absorption due to C = O groups deformation within the alkyl groups of the lignin side chains (50). Our results showed that the absorption peak for C = O groups reduced after pretreatment since the alkali hydrolysis reaction may cause partial lignin structure to change from raw rice straw. Collectively, the FTIR spectra data further confirmed that the CSL plus urea-alkali pretreatment degraded a significant portion of the lignin, cellulose and hemicellulose.

#### Changes in cellulose crystallinity

**Figure 2:**
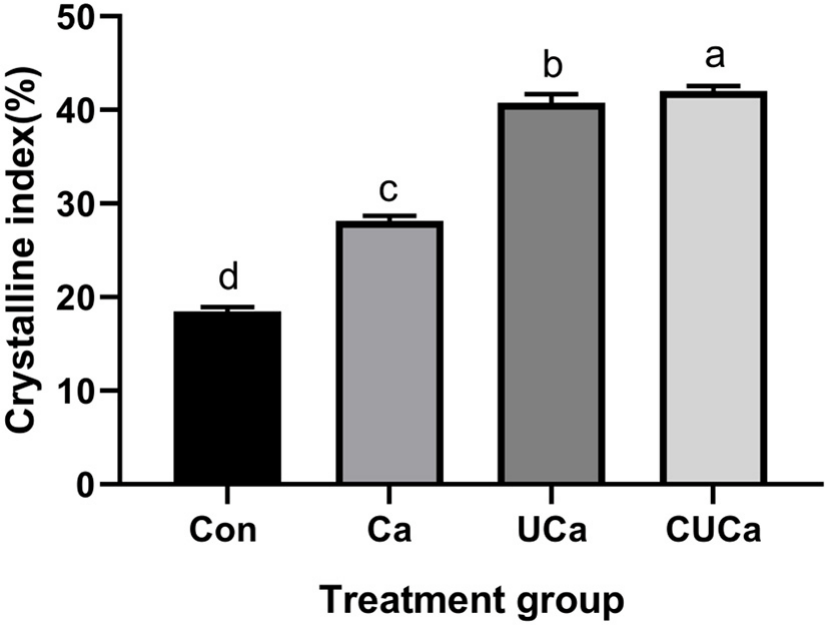
Effect of CSL plus urea-alkali pretreatment on the cellulose crystalline index (CrI) of rice straw. Con: no pretreatment for control group, Ca: 4% CaO, UCa: 2.5% urea + 4% CaO, CUCa: 9% CSL + 2.5% urea + 4% CaO. Different superscript letters a, b, c, and d indicates significantly different values (*P* < 0.05) in different groups, and the same or no letters indicate insignificant differences (*P* > 0.05).

**Figure 3:**
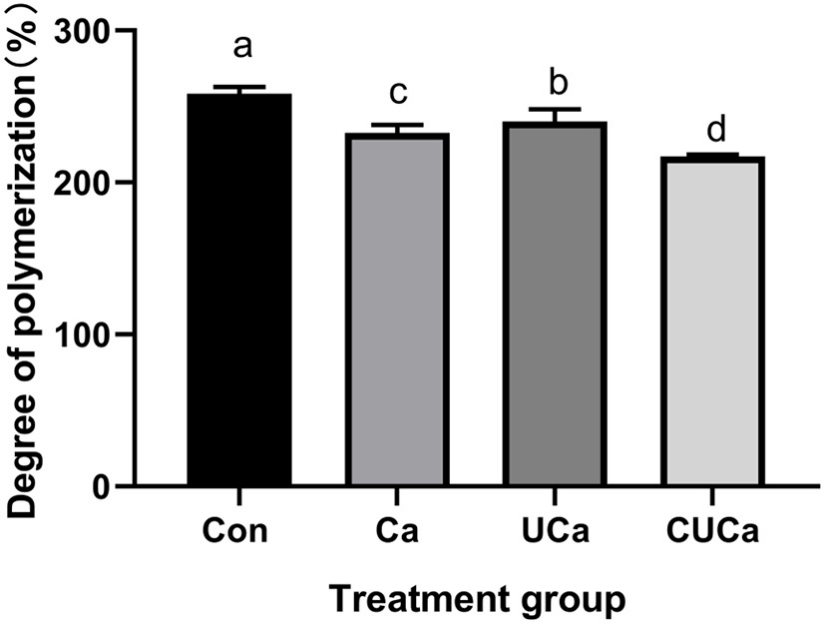
Effect of CSL plus urea-alkali pretreatment on the cellulose degree of polymerization (DP) of rice straw. Con: no pretreatment for control group, Ca: 4% CaO, UCa: 2.5% urea + 4% CaO, CUCa: 9% CSL + 2.5% urea + 4% CaO. Different superscript letters a, b, c, and d indicates significantly different values (*P* < 0.05) in different groups, and the same or no letters indicate insignificant differences (*P* > 0.05).

Lignocellulosic biomass includes amorphous components (hemicellulose and lignin) and crystalline components (cellulose). Chemical pretreatment modified the crystal structure followed by decreases in the CrI of lignocellulosic biomass. However, this decrease may be affected by hemicellulose content. In the current study, the analysis of X-ray diffraction revealed that the CSL plus urea-alkali pretreatment increased (*P* < 0.05) the CrI of the rice straw (Figure 2). The higher CrI of the CSL plus urea-alkali pretreatment was probably ascribed to the removal of non-cellulose components and thus the increase in the cellulose proportion. These findings corroborate those of Gu et al. (51) who also showed increases in CrI after Ca(OH)_2_ pretreated rice straw. It should be noted that, even though the CSL plus urea-alkali pretreatment had a higher CrI than untreated rice straw, while its degradation was still higher than the later as demonstrated by the *in vitro* digestibility (Table 2). Therefore, CrI is not the only criteria for evaluating straw digestibility, and high crystallinity does not necessarily lead to poor digestibility. This is because the CSL plus urea-alkali pretreatment had reduced the polymerization (Figure 3) of lignin and broke the bonds between lignin and cellulose so that ruminal microbes had better access to and colonization on cellulose.

**Table 2:** Effect of CSL plus urea-alkali pretreatment on digestibility of DM, NDF, and ADF of rice straw *in vitro* after 15 days of storage.

**Items**	**Treatment**				**SEM**	***P*-value**
	**Con**	**Ca**	**UCa**	**CUCa**		
IVDMD, %	53.00^d^	55.95^c^	60.65^b^	65.98^a^	0.782	<0.001
IVNDFD, %	46.59^c^	48.99^c^	53.22^b^	58.88^a^	0.923	<0.001
IVADFD, %	45.07^c^	41.94^d^	49.72^b^	53.08^a^	0.991	<0.001

Different superscript letters a, b, c, and d indicates significantly different values (P <0.05) across-rows, and the same or no letters indicate insignificant differences (P > 0.05).

IVDMD, In vitro DM degradability; IVNDFD, In vitro NDF degradability; IVADFD, In vitro ADF degradability; SEM, standard error of means.

Con: no pretreatment for control group, Ca: 4% CaO, UCa: 2.5% urea + 4% CaO, CUCa: 9% CSL + 2.5% urea + 4% CaO.

#### Alteration of cellulose degree of polymerization

Cellulose degree of polymerization (DP) is an important parameter in studying of cellulose structural features (52). Consistently, it has been reported that cellulose DPs are the negative factors affecting biomass enzymatic digestibility (31). In the current study, the large reduction (*P* < 0.001) of the DP of crystalline cellulose by the CSL plus urea-alkali pretreatment (Figure 3), indicated that enhancing biomass enzymatic saccharification in rice straw. The reduction in cellulose DP significantly improved biomass enzymatic hydrolysis due to the increase in the number of cellulose chain-reducing ends (53). This is also consistent with the *in vitro* digestibility results of rice straw obtained from the current research.

#### Changes in morphological structure of rice straw

**Figure 4:**
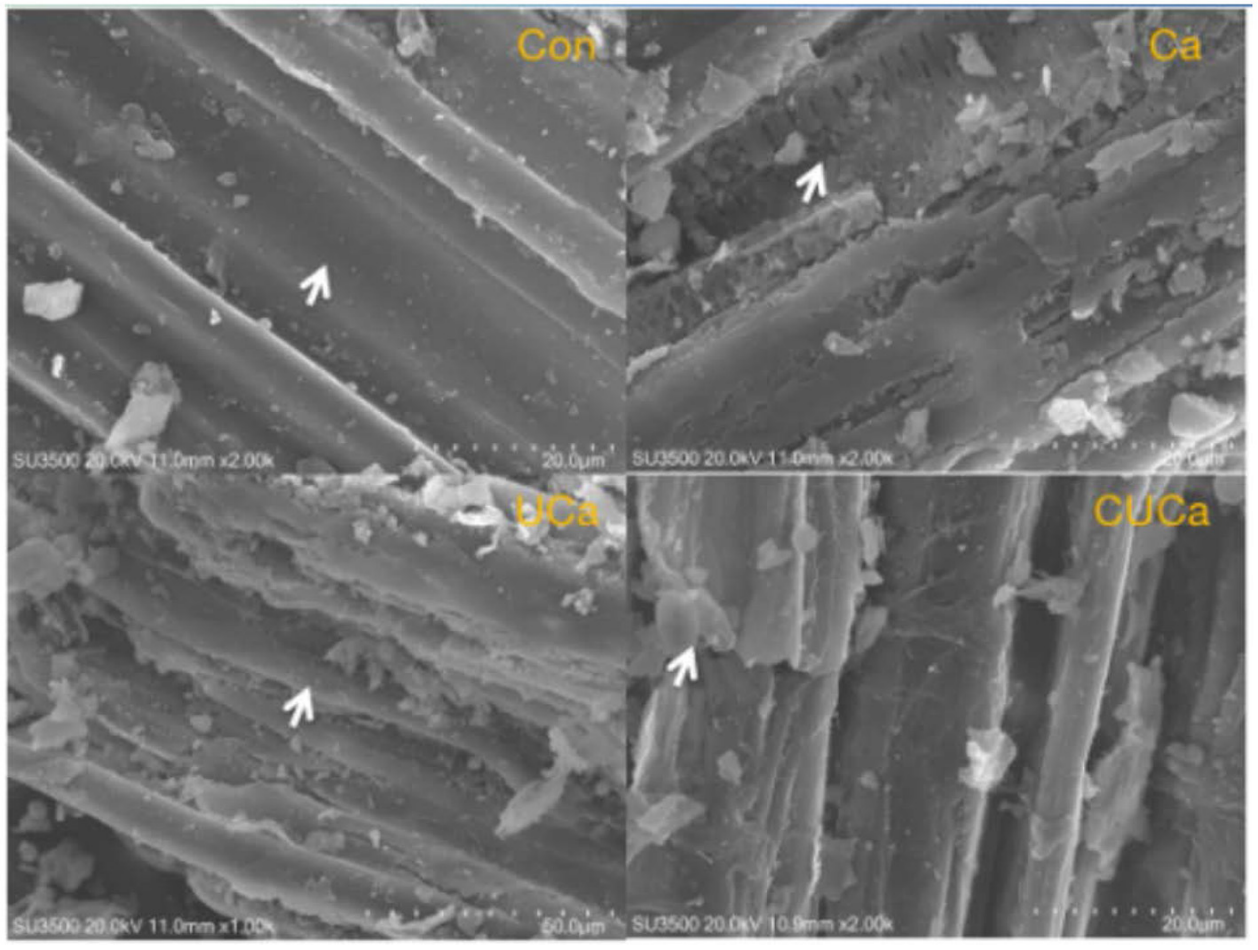
SEM imagines of biomass residues obtained from pretreatment with CSL plus urea-alkali. Con: no pretreatment for control group, Ca: 4% CaO, UCa: 2.5% urea + 4% CaO, CUCa: 9% CSL + 2.5% urea + 4% CaO. Sample (Ca, UCa, CUCa) showing a coarse surface indicated as arrow, and sample Con displaying a flat face.

Based on SEM micrographs (Figure 4), the CSL plus urea-alkali pretreatment exhibited several significant alterations in the surface of rice straw. The untreated rice straw showed a compact and smooth surface structure. These surface features would hinder rumen microbial attachment and colonization of rice straw. However, after the CSL plus urea-alkali pretreatment, the rice straw surface obvious became rougher and more disordered. This was probably due to the partial dissolving of the hemicellulose structure by alkalinity. Similar structural changes were found in other lignocellulosic biomass subjected to alkali pretreatment (54). Furthermore, as the surface structure was damaged by CSL plus urea-alkali pretreatment, the internal contents were exposed, which would promote microbial colonization and enhance digestion.

### Enzymatic hydrolysis of rice straw after CSL plus urea-alkali pretreatment

**Figure 5:**
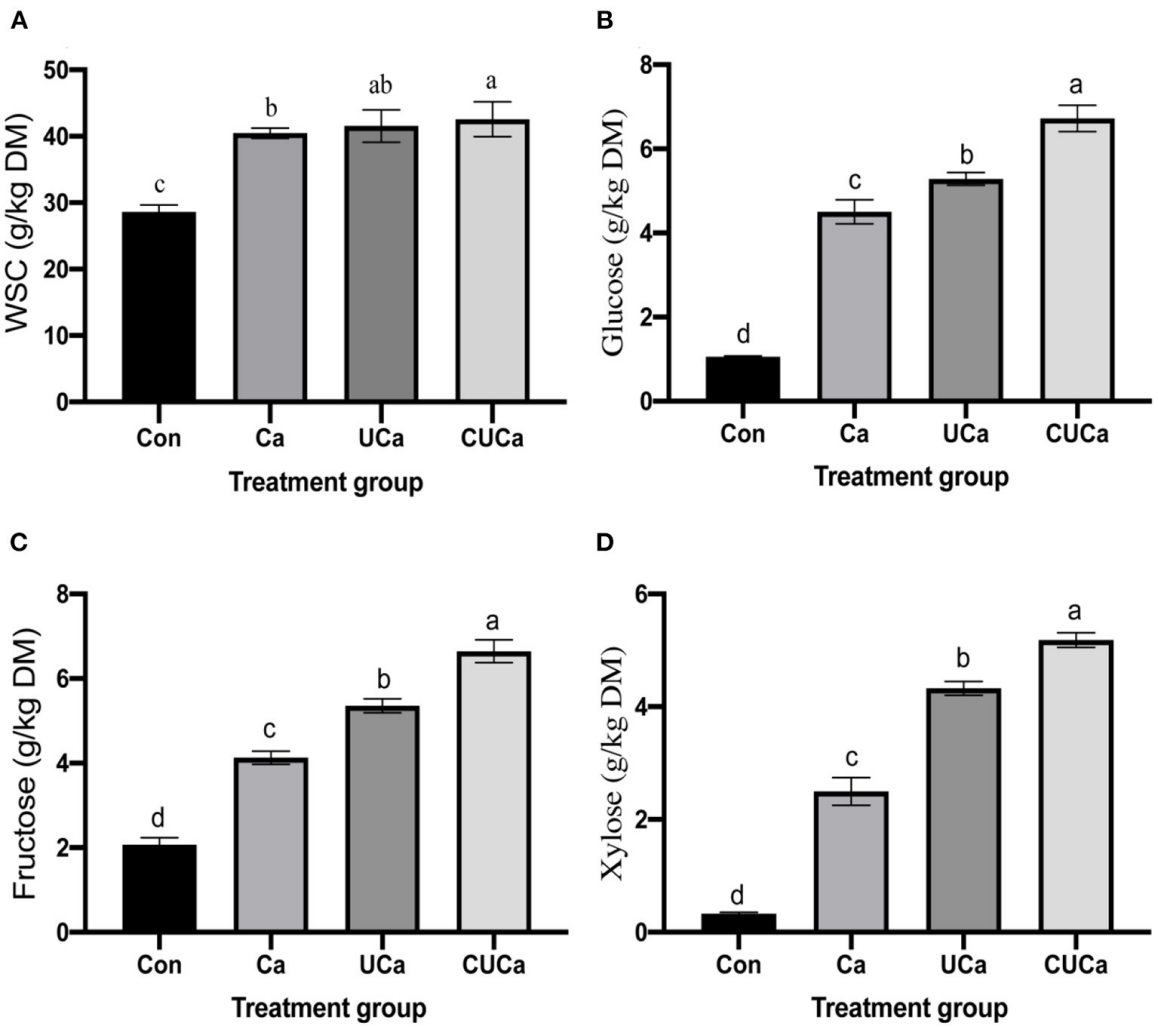
Effect of CSL plus urea-alkali pretreatment on **(A)** water soluble carbohydrates (WSC) yield, **(B)** glucose, **(C)** fructose, and **(D)** xylose of rice straw. Con: no pretreatment for control group, Ca: 4% CaO, UCa: 2.5% urea + 4% CaO, CUCa: 9% CSL + 2.5% urea + 4% CaO. Different superscript letters a, b, c, and d indicates significantly different values (*P* < 0.05) in different groups, and the same or no letters indicate insignificant differences (*P* > 0.05).

The main purpose of the pretreatment of lignocellulose is to remove the hemicellulose and lignin and enhance its porosity and surface area followed by improvement in its saccharification efficiency and the utilization efficiency of biomass during microbial fermentation (55). This revealed that the CSL plus urea-alkali pretreatment have satisfactorily fulfilled the purpose. To test the efficiency of pretreatment, enzymatic saccharification of the untreated and pretreated rice straw samples was investigated, and the results are shown in Figures 5A,D. The CSL plus urea-alkali pretreatment significantly affected the contents of WSC, glucose, fructose and xylose (*P* < 0.05). The much higher concentration of WSC released from the pretreated rice straw samples (Figure 5A) could be attributed to the higher porosity and surface area, and lower NDF and ADF content. Specifically, the WSC yield of the untreated rice straw (Con) at 72 h was only 28.60 g/kg DM, while it was increased up to 40.46, 41.55, and 42.57 g/kg DM by Ca, UCa, and CUCa pretreatments, respectively. It has been reported that continuous 5% CaO pretreatment reeds could lead to the highest ethanol production reaching 19% with almost complete sugar-to-ethanol conversion (56). This could be due to alteration of cell wall structure matrix as indicated by their structure analysis, and increase in the surface area and porosity of the rice straw samples by removal of hemicellulose and lignin, which result in released more WSC from rice straw (8).

The concentration of larger fructose and xylose content in Ca, UCa, and CUCa (Figures 5C,D) indicated that the hemicellulose fraction of rice straw was effectively hydrolyzed to xylose by CSL plus urea-alkali. Xylose, as a typical pentose sugar, originates from the degradation of hemicellulose (57). Figure 5B quantitatively showed the enhancement in glucose by pretreatment.

According to the results of changes in WSC concentration, the release rate of WSC was faster in CSL plus urea alkali pretreatment than in untreated straw. Similar changes were found in Ca(OH)_2_ treatment of rice straw (51).

### Rumen fermentation of rice straw after CSL plus urea-alkali pretreatment

Compared with the untreated rice straw, the UCa and CUCa pretreatment had a higher (*P* < 0.001) IVDMD, IVNDFD, and IVADFD (Table 2), which indicated that the quality was improved of rice straw after UCa and CUCa pretreatment. There may be two reasons for the increased *in vitro* nutrient digestibility of rice straw after UCa and CUCa pretreatment. First, the pretreatment destroys the lignocellulose structure of rice straw and reduces the recalcitrance. Secondly, due to the change of rice straw structure, the surface area and porosity of rice straw increased, which provided more colonization sites for rumen microorganisms and improved the degradation efficiency. Similarly, a study had shown that higher the IVDMD value, the better will be the quality of roughages (58).

The gas production changes during *in vitro* culture process are mentioned in Supplementary Figure S1. We found that the gas production of each group gradually increased with the extension of the *in vitro* culture time. Compared with the untreated rice straw, CSL plus urea-alkali pretreatment had a higher (*P* < 0.05) GP_48h_ (Table 3). Gas production has been found to be linked with the feed chemical composition; the easily fermentable material produces more gas and faster than the less fermentable material (59). The more gas production from Ca, UCa, and CUCa suggested that the CSL plus urea-alkali pretreatment increased the rice straw digestibility by disrupting the cell wall structure of rice straw and providing an increased surface for rumen microbial colonization.

**Table 3:** Effect of CSL plus urea-alkali pretreatment on gas production characteristics of rice straw.

**Items**	**Treatment**				**SEM**	***P*-value**
	**Con**	**Ca**	**UCa**	**CUCa**		
GP_48h_ (mL/g, DM)	30.32^c^	41.53^b^	44.53^b^	55.65^a^	1.294	<0.001
GP_max_ (mL/g, DM)	44.44^b^	61.43^a^	58.53^a^	61.81^a^	2.833	0.001
C, %/h	1.28^a^	1.12^b^	1.23^ab^	1.17^ab^	0.042	0.035
Half-time, h	32.61^a^	24.03^b^	18.43^c^	18.92^c^	0.913	<0.001

Different superscript letters a, b, and c indicate significantly different values (P <0.05) across-rows, and the same or no letters indicate insignificant differences (P > 0.05). Con: no pretreatment for control group, Ca: 4% CaO, UCa: 2.5% urea + 4% CaO, CUCa: 9% CSL + 2.5% urea + 4% CaO.

SEM, standard error of means.

GP_48h_: gas production in 48 h, GP_max_: ideal maximum gas production, C: the constant fractional rate (%/h); half-time: the time needed when gas production is 1/2 of the maximum.

The CSL plus urea-alkali pretreatment produced a higher (*P* < 0.05) concentration of butyrate, acetate, and total VFA than the untreated rice straw (Table 4). The increased WSC release from the CSL plus urea-alkali pretreatment mirrors the higher production of VFA. The improved performance (i.e., increased VFA and gas production and release of WSC) results from the *in vitro* fermentation of CSL plus urea-alkali pretreatment clearly suggests that the CSL plus urea-alkali pretreatment improve the metabolic activities of the ruminal microbiome and can be used as forage for ruminants.

**Table 4:** Effect of CSL plus urea-alkali pretreatment on *in vitro* rumen fermentation parameters of rice straw.

**Items**	**Treatment**				**SEM**	***P*-value**
	**Con**	**Ca**	**UCa**	**CUCa**		
pH	6.80	6.72	6.69	6.69	0.012	0.385
Acetic acid (mM/L)	40.31^d^	47.31^b^	44.48^c^	50.58^a^	0.783	<0.001
Propionic acid (mM/L)	18.11^b^	18.10^b^	18.90^ab^	20.00^a^	0.434	0.016
Butyric acid (mM/L)	5.53^b^	6.63^a^	6.89^a^	7.14^a^	0.274	0.002
Total volatile fatty acid (mM/L)	65.56^c^	75.86^b^	73.93^b^	81.78^a^	1.272	<0.001

Different superscript letters a, b, c, and d indicate significantly different values (P <0.05) across-rows, and the same or no letters indicate insignificant differences (P > 0.05).

Con: no pretreatment for control group, Ca: 4% CaO, UCa: 2.5% urea + 4% CaO, CUCa: 9% CSL + 2.5% urea + 4% CaO.

SEM, standard error of means.

### Effect of CSL plus urea-alkali pretreatment on microbial colonization

**Figure 6:**
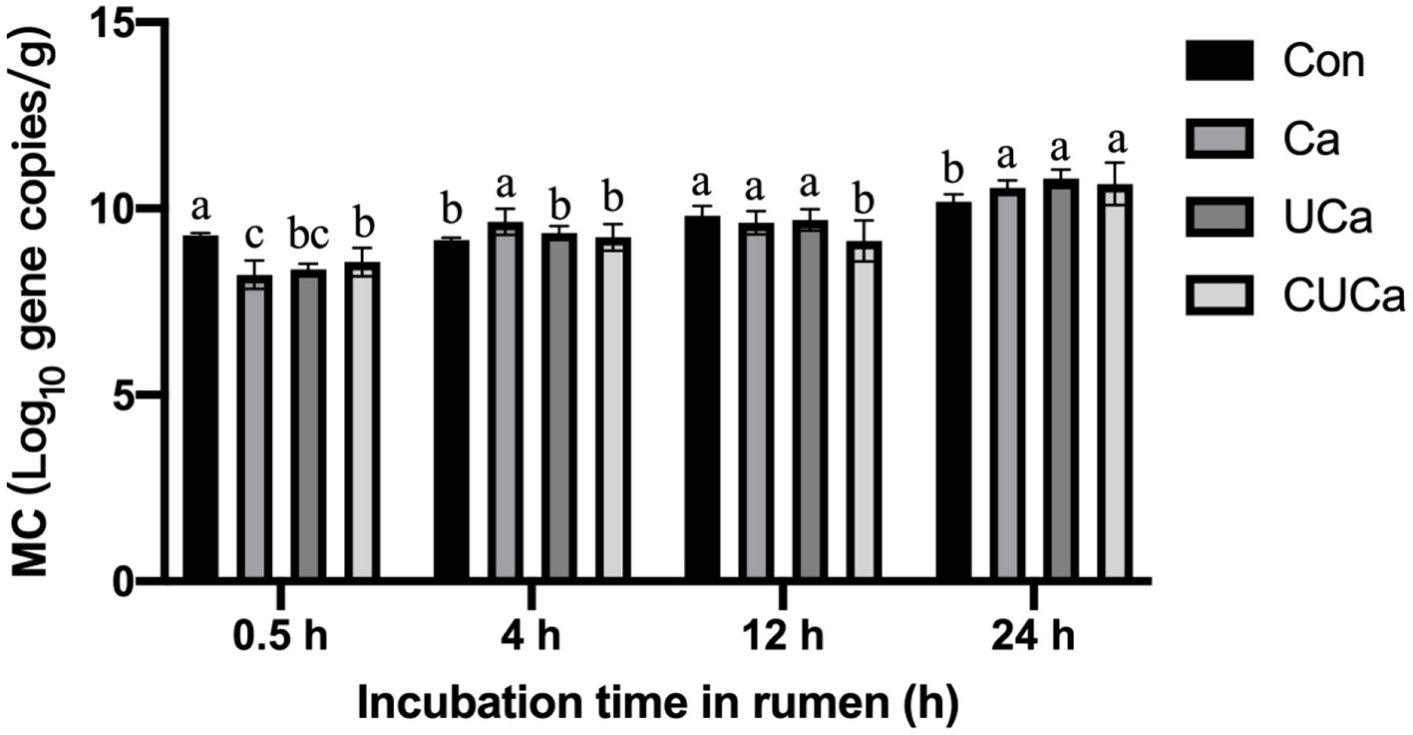
Microbial colonization (MC) (log_10_ gene copies/g of undigested rice straw) of rice straw following incubation for up to 24 h. Con: no pretreatment for control group, Ca: 4% CaO, UCa: 2.5% urea + 4% CaO, CUCa: 9% CSL + 2.5% urea + 4% CaO. Different superscript letters a, b, and c indicates significantly different values (*P* < 0.05) in different groups, and the same or no letters indicate insignificant differences (*P* > 0.05).

As estimated with an analysis of real-time PCR by examine the total copy number of bacterial 16S rRNA genes, the CSL plus urea-alkali pretreatment markedly increased (*P* < 0.05) microbial colonization on the surface of the Ca, UCa and CUCa (Figure 6). After 0.5 h of rumen incubation, fewer microorganisms were found on the surface of the rice straw that had received CSL plus urea-alkali pretreatment than on untreated rice straw. In addition, more microorganisms were observed on the surface of CSL and rice straw that had been pretreated with urea-alkali compared to un-treated following incubation for 0.5, 4, 12, and 24 h. Microbial colonization on fibrous feed particles was affected by substrate characteristics and WSC (60). Obviously, the CSL plus urea-alkali pretreatment increased the porosity of rice straw and the surface area of microbial colonization (8). The increase of microbial colonization on CSL plus urea-alkali pretreatment surface was consistent with the change in surface morphology and the increase of *in vitro* gas production.

### Effect of CSL plus urea-alkali pretreatment on bacterial community structure

Alpha diversity analysis of attached bacteria (Supplementary Figure S2) shown by Chao 1 index, the Ca and UCa pretreatment had significant accelerative (*P* < 0.001) effects on bacterial community richness compared with untreated rice straw at 0.5 h incubation, CUCa pretreatment had significant richness (*P* < 0.05) at 4 h compared with untreated rice straw. Diverse microbial compositions were detected among groups in both phylum (Supplementary Figure S3a) and family (Supplementary Figure S3b) levels. At the phylum levels, the relative abundance of *Firmicutes* gradually decreased with the prolongation of incubation time. The relative abundance of *Firmicutes* in Ca, UCa, and CUCa groups was higher than that in control group at the beginning of incubation. When incubated for 24 h, the relative abundance of *Firmicutes* among the groups was basically the same. The relative abundance of *Bacteroidota* showed the opposite trend, with the incubation time increasing, the relative abundance of *Bacteroidota* in the Ca, UCa, and CUCa groups was lower than that in the control group at the beginning of incubation. When incubated for 24 h, the relative abundance of *Bacteroidota* remained basically the same among the groups. At the family levels, the relative abundance of *Camobacteriaceae* changed significantly. At the beginning of incubation, the relative abundance of *Camobacteriaceae* in all treatment groups was higher than that in the control group. PCoA based on weighted unifrac distance (Supplementary Figure S4) suggests that a dispersed data points on plots of all the four groups in 0.5 h (*R*^2^ = 0.3611, *P* = 0.014), 4 h (*R*^2^ = 0.2407, *P* = 0.085), 12 h (*R*^2^ = 0.3642, *P* = 0.034), and 24 h (*R*^2^ = 0.3765, *P* = 0.011). Notably, LEfSe analysis showed that the *Carnobacteriaceae*, Aerococcaceae, *Staphylococcaceae, Carnobacterium, Aerococcus, Jeotgalibaca, Facklamia*, and *Staphylococcus* were enriched post CSL plus urea-alkali pretreatment (Supplementary Figure S5). Importantly, diet preference significantly affected the rumen microbial diversity of healthy dairy cows (61). The fistulated cows in our study were fed same diet, and therefore detected the microorganisms observed on the surface of the rice straw were determined by the difference in pretreatment. To understand how did the CSL plus urea-alkali pretreatment affect fecal microbiome (taxonomic and structure) accurately, we compared the bacterial compositions among Con, Ca, UCa, and CUCa groups, the relative abundance of total bacterial genus enrichment in Ca and UCa in 0.5 h and in CUCa in 4 h, separately. Meanwhile, obvious alterations of the microbiome structure were detected. The attachment of feed rumen microbial populations is notably a critical step phase in the process of rumen digestion and fermentation (62). In addition, the attachment of bacteria with fiber degradation ability can improve the degradation of the feed in rumen (20). Here, the finding of this study indicated that the CSL plus urea-alkali pretreatment promoted rice straw degradability by altering the compositions of rumen microbiota colonized on the surface of rice straw, including a severe increase in the abundance of *Camobacteriaceae, Aerococcaceae, Prevotellaceae, Staphylococcaceae*. Indeed, *Camobacteriaceae* has been reported to correlate with the destruction of lignocellulosic cell walls (63). Besides, *Staphylococcaceae* and *Prevotellaceae* play a key role in lignocellulose degradation (64, 65). Interestingly, in our findings, we observed that CSL plays an important role in inducing colonization of *Camobacteriaceae, Aerococcaceae*, and *Staphylococcaceae*, because the colonization of these bacteria occurs only in UCa or CUCa and is not found in the Ca group. Thus, future studies are needed to verify these bacterial functions.

### Link between rumen bacterial attachment on the surface of rice straw and environmental factors

**Figure 7:**
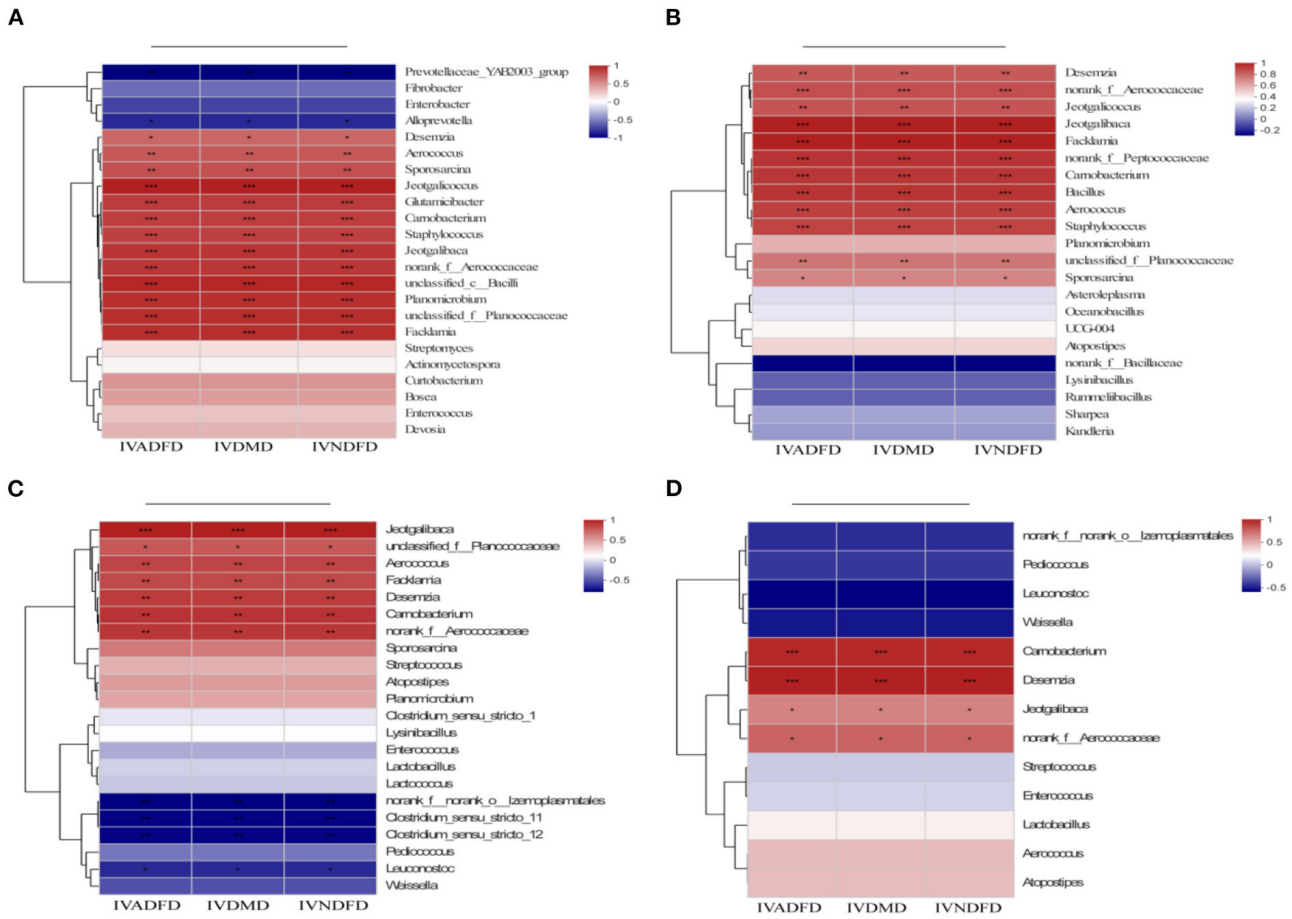
Correlations heatmap of top 30 genera in colonization on surface of rice straw during incubation in rumen 0.5 h **(A)**, 4 h **(B)**, 12 h **(C)**, and 24 h **(D)** and environmental factors. Con: no pretreatment for control group, Ca: 4% Cao, UCa: 2.5% urea + CaO, CUCa: 9% CSL + 2.5% urea + 4% CaO. *, **, and *** indicated the significant correlations at *P* < 0.05, 0.01, and 0.001. IVDMD, *in vitro* dry matter degradability; IVNDFD, *in vitro* neutral detergent fiber degradability.

The spearman correlation heatmap of the top genera and environmental parameters was used to further evaluate the association between attachment of bacteria on the surface of rice straw following CSL plus urea-alkali treatment of rumen flora (IVDMD, IVNDFD and IVADFD). As showed in Figures 7A,D, the *Alloprevotella* and *Prevotellaceae_YAB2003_group* after 0.5 h (Figure 7A) and *norank_f_O_lzemopasmatales, Clostridium_sensu_stricto_11, Clostridium_sensu_stricto_12, Pediococcus* and *Leuconostoc* (Figure 7C) after 12 h of incubation in the rumen had a strong negative correlation with IVDMD, IVNDFD and IVADFD of rice straw (*P* < 0.05). Notably, these bacteria were enriched in the Con group. Indeed, *Alloprevotella, Prevotellaceae_YAB2003_group, Clostridium_sensu_stricto_12* and *Pediococcus* have been reported to correlate with low digestibility of forages (66). By contrast, the *Desemzia, Aerococcus, Carnobacterium, Staphylococcus, Jeotgalibaca, Jeotgalicoccus, norank_f__Aerococcaceae, Glutamicibacter, Facklamia, Sporosarcina, unclassified_c__Bacilli* and *Planomicrobium* after 0.5 h (Figure 7A) and the *Carnobacterium, Staphylococcus, Aerococcus, Facklamia, norank_f__Peptococcaceae, Jeotgalibaca* and *norank_f__Aerococcaceae* after 4 h (Figure 7B) and the *Carnobacteriaceae, Carnobacterium, Desemzia, Planococcaceae, Aerococcaceae*, and *Aerococcus* after 12 (Figure 7C) and *Carnobacterium, Desemzia, Jeotgalibaca and norank_f__Aerococcaceae* (Figure 7D) after 24 h of incubation in the rumen had positive correlation (*P* < 0.05) with IVDMD, IVNDFD, and IVADFD of rice straw. Importantly, these bacteria were enriched with rice straw of the CSL plus urea-alkali pretreatment. Actually, these bacteria mainly belonged to *Firmicutes*, which are enriched for genes associated with the lignocellulosic polymers degradation and the fermentation of degraded products into short-chain volatile fatty acids (62), conducive to the lignocellulose degradation. Interestingly, the *Carnobacteriaceae* were always attached to the CUCa during the entire incubation process in the rumen.

## Conclusions

Altogether, we concluded that the CSL plus urea-alkali pretreatment of rice straw was effective in enhancing rice straw degradation by the ruminal microbiome. The CSL plus urea-alkali pretreatment improved the enzymatic hydrolysis and surface area of rice straw by destructing its structure. Furthermore, the enhanced fiber digestion, microbial colonization, and fermentation was documented in response to CSL plus urea-alkali pretreatment. Importantly, the CSL plus urea-alkali pretreatment could directional induction of some bacterial attachment such as *Carnobacterium* and *Staphylococcus*, which have strong fiber degrading ability. Finally, we recommended that combination of 9% corn steep liquor, 2.5% urea and 4% CaO can be utilized to develop large-scale processes for the improvement of the nutritional value of rice straw as feedstock for biofuel production or as forage for ruminants.

## Data availability statement

The datasets presented in this study can be found in online repositories. The names of the repository/repositories and accession number(s) can be found in the article/Supplementary material.

## Ethics statement

The animal study was reviewed and approved by the Animal Care Committee of the School of Animal Science and Technology, China Agricultural University (Protocol Number: 2013-5-LZ) has reviewed and approved all the animals used in this study.

## Author contributions

ZC and YM mainly designed this experiment. YM conducted the experiments. Data were collected and analyzed by YM and XC. JX, SL, JW, and GA collected the samples and performed the analysis of samples. The manuscript was mainly written by ZC and YM and edited by MZ. All authors contributed to the article and approved the submitted version.

## Funding

This work was financially supported by the 2115 Talent Development Program of China Agricultural University and Gushi professor workstation.

## Conflict of interest

The authors declare that the research was conducted in the absence of any commercial or financial relationships that could be construed as a potential conflict of interest.

## Publisher's note

All claims expressed in this article are solely those of the authors and do not necessarily represent those of their affiliated organizations, or those of the publisher, the editors and the reviewers. Any product that may be evaluated in this article, or claim that may be made by its manufacturer, is not guaranteed or endorsed by the publisher.
