# Supplementary material for: Biodegradation and hydrolysis of rice straw with corn steep liquor and urea-alkali pretreatment
Source: Front Nutr. 2022 Aug 4;9:989239. doi: 10.3389/fnut.2022.989239 (PMC9387106; doi:10.3389/fnut.2022.989239)
Supplement: Supplementary file 1 [file Data_Sheet_1.pdf]

Biodegradation and hydrolysis of rice straw with corn steep liquor and urea-alkali pretreatment

Yulin Ma[a], Xu Chen[a], Muhammad Zahoor Khan[a], Jianxin Xiao[a], Shuai Liu[a], Jingjun Wang[a], Gibson Maswayi Alugongo[a], Zhijun Cao[a]*

[a]State Key Laboratory of Animal Nutrition, College of Animal Science and Technology, China Agricultural University, Beijing 100193, PR China

**Correspondence: caozhijun@cau.edu.cn; Tel.: +86-10-62733746*

Supplemental Table 1. Basic diet composition and nutrient value (DM basis %)

**Items**	**Content**	**Nutrient levels[2]**	**Content**
Ingredients		NE_L（MJ/Kg）	5.68
Chinese wildrye	6.38	CP	15.01
Alfalfa hay	20.31	EE	3.4
Oat hay	5.58	NDF	41.03
Wheat	1.76	ADF	26.69
Corn silage	24.48	Ca	0.55
Corn	3.66	P	0.39
Steam-flaked corn	13.52		
Soybean meal	6.55		
Extruded soybean	3.76		
Soybean hull	3.00		
Cottonseed	3.41		
Molasses	3.80		
Rumen-pass fatty acid	1.20		
Yeast powder	0.20		

Mycotoxin re-movement agent	0.06
NaCl	0.31
Limestone	0.32
$Ca(HCO_3)_2$	0.34
$NaHPO_3$	0.67
$KHCO_3$	0.26
Premix[1]	0.31
MgO	0.12

[1)]Each kilogram of premix contains vitamin A 1000,000 IU/kg, vitamin D 3280,000 IU/kg, vitamin E 10,000 IU/kg, 1000 mg/kg nicotinic acid, 0.6 mg/kg copper, 1.2 mg/kg zinc, 2.2 mg/kg manganese, 76 mg/kg iodine, 5.5 mg/kg selenium, 29 mg/kg cobalt.

[2)] NE_L is a calculated value, while the other nutrient levels were measured values.
