# Supplementary material for: Biodegradation and hydrolysis of rice straw with corn steep liquor and urea-alkali pretreatment
Source: Front Nutr. 2022 Aug 4;9:989239. doi: 10.3389/fnut.2022.989239 (PMC9387106; doi:10.3389/fnut.2022.989239)
Supplement: Supplementary file 2 [file Data_Sheet_2.pdf]

Biodegradation and hydrolysis of rice straw with corn steep liquor and urea-alkali pretreatment

Yulin Ma[a], Xu Chen[a], Muhammad Zahoor Khan[a], Jianxin Xiao[a], Shuai Liu[a], Jingjun Wang[a], Gibson Maswayi Alugongo[a], Zhijun Cao[a]*

[a]*State Key Laboratory of Animal Nutrition, College of Animal Science and Technology, China Agricultural University, Beijing 100193, PR China;*

**Correspondence: caozhijun@cau.edu.cn; Tel.: +86-10-62733746*

Supplementary Information

Figure S1. Cumulative gas production curve at 48 h of different groups. Con: no pretreatment for control group, Ca: 4% Cao, UCa: 2.5% urea + CaO, CUCa: 9% Corn steep liquor + 2.5% urea + 4% CaO.

Figure S2. α-diversity of different groups after incubation in rumen 0.5 h (A), 4 h (B), 12 h (C) and 24 h (D) by chao1 index. Con: no pretreatment for control group, Ca: 4% Cao, UCa: 2.5% urea + CaO, CUCa: 9% Corn steep liquor + 2.5% urea + 4% CaO. Data were mean±SEM. P values were determined using the t-test. *P⩽0.05, **P⩽0.01.

Figure S3. The relative abundance of colonization bacterial on surface of rice straw of different groups after incubation in rumen 0.5 h (A), 4 h (B), 12 h (C) and 24 h (D) at

phylum (**a**) and family (**b**) level presented in 99.5% of the community. Ca: 4% Cao, UCa: 2.5% urea + CaO, CUCa: 9% Corn steep liquor + 2.5% urea + 4% CaO.

Figure S4. PCoA plots based on the weighted UniFrac distance matrix upon different groups after incubation in rumen 0.5 h (A), 4 h (B), 12 h (C) and 24 h (D). The data were assessed using PERMANOVA analysis, with 999 permutations. Ca: 4% Cao, UCa: 2.5% urea + CaO, CUCa: 9% Corn steep liquor + 2.5% urea + 4% CaO.

Figure S5. The enriched rumen microbiota taxa was shown by LEfSe (linear discriminant analysis (LDA) coupled with effect size measurements) of different groups after incubation in rumen 0.5 h (A), 4 h (B), 12 h (C) and 24 h (D). Ca: 4% Cao, UCa: 2.5% urea + CaO, CUCa: 9% Corn steep liquor + 2.5% urea + 4% CaO.
