# Supplementary figures and images for: Biodegradation and hydrolysis of rice straw with corn steep liquor and urea-alkali pretreatment
Source: Front Nutr. 2022 Aug 4;9:989239. doi: 10.3389/fnut.2022.989239 (PMC9387106; doi:10.3389/fnut.2022.989239)

Figure S1

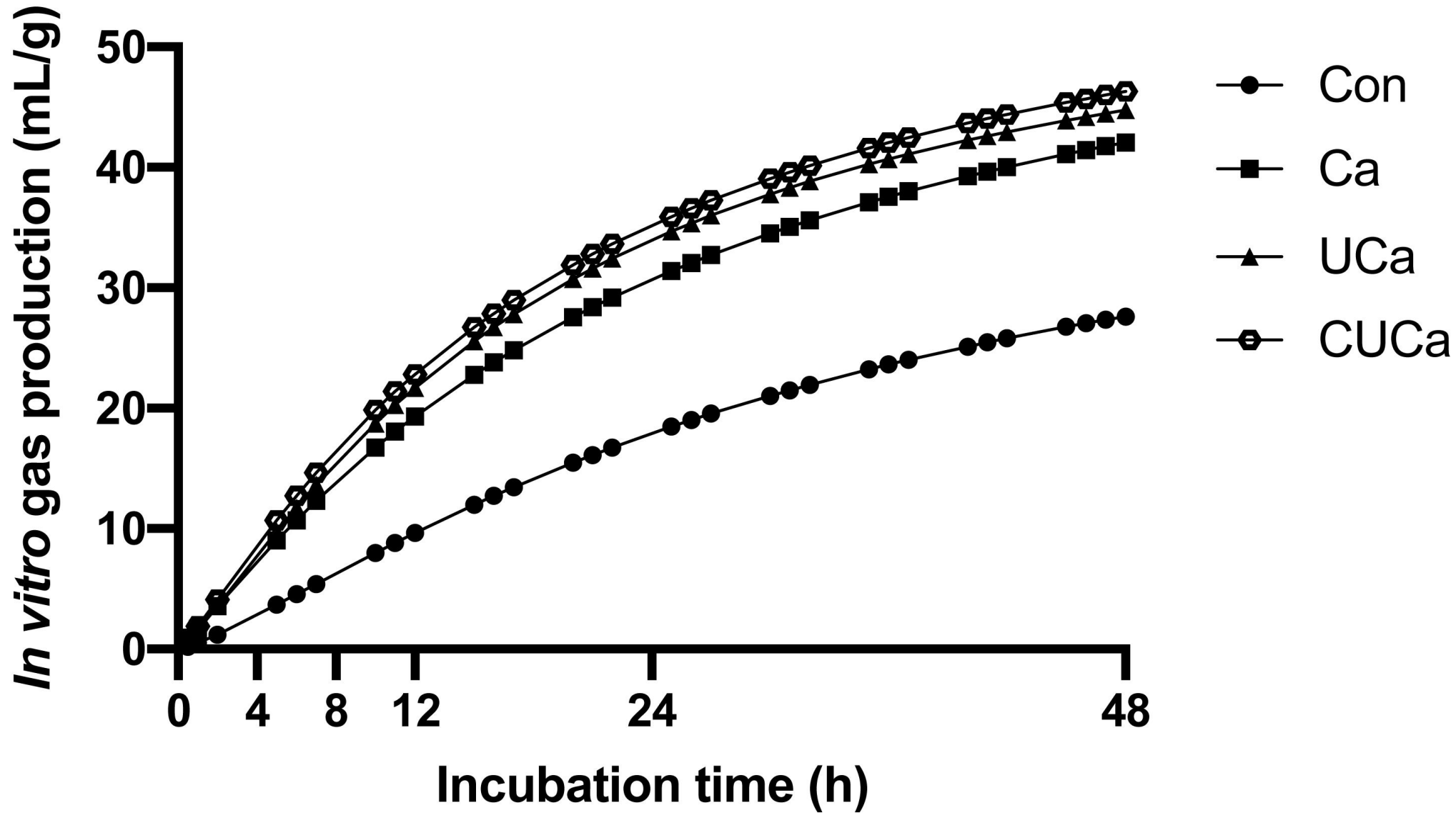

Supplement: Supplementary file 3 [file Image_1.pdf]

Figure S2

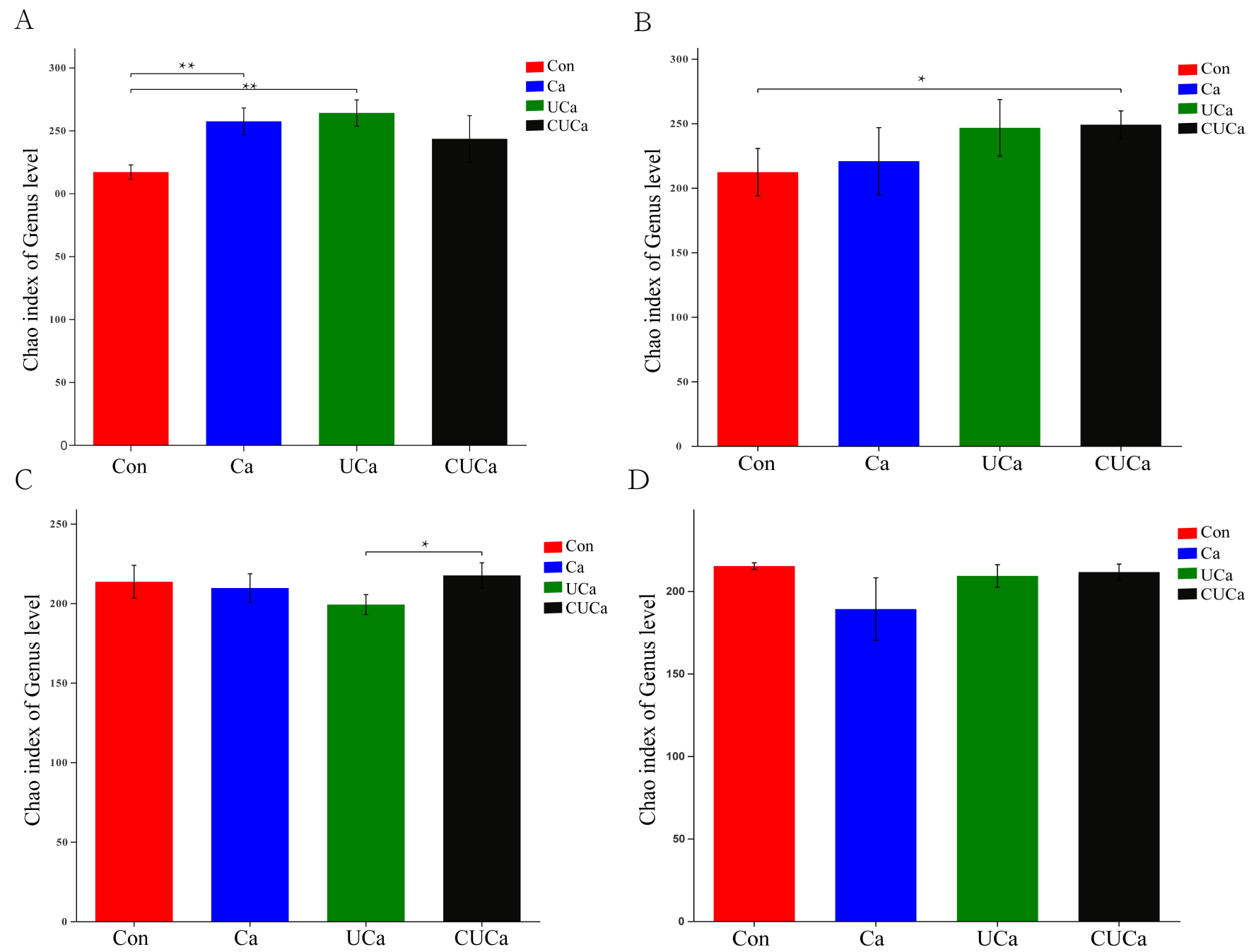

Supplement: Supplementary file 4 [file Image_2.pdf]

Figure S3

b

A

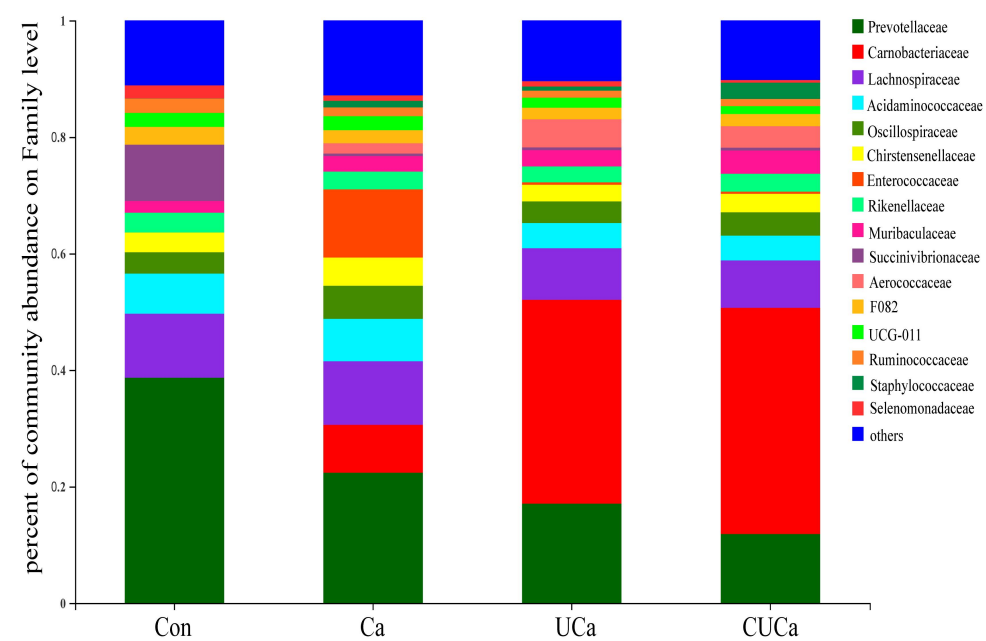

B

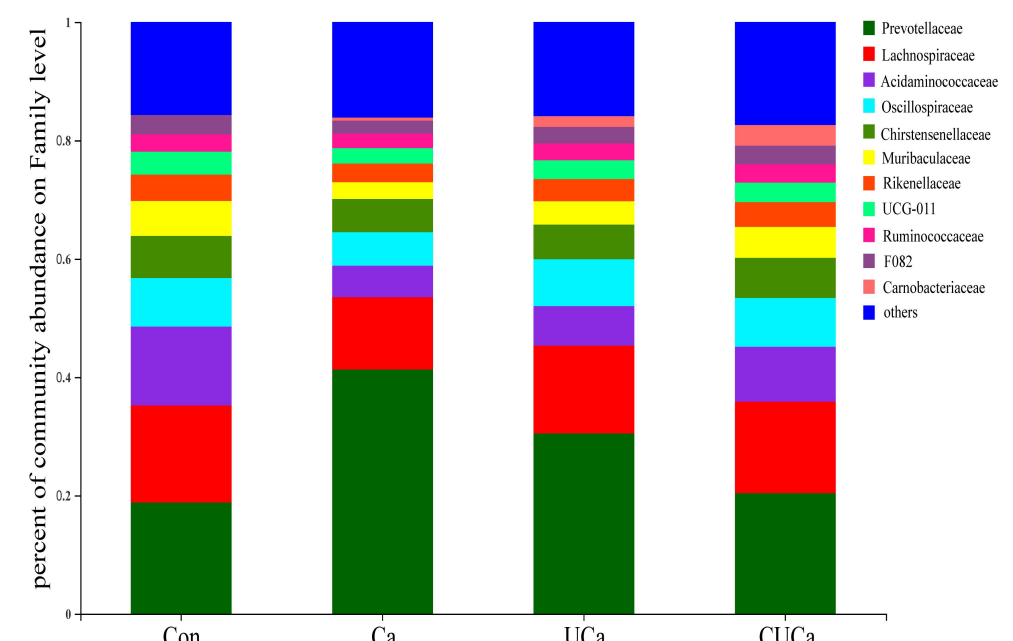

C

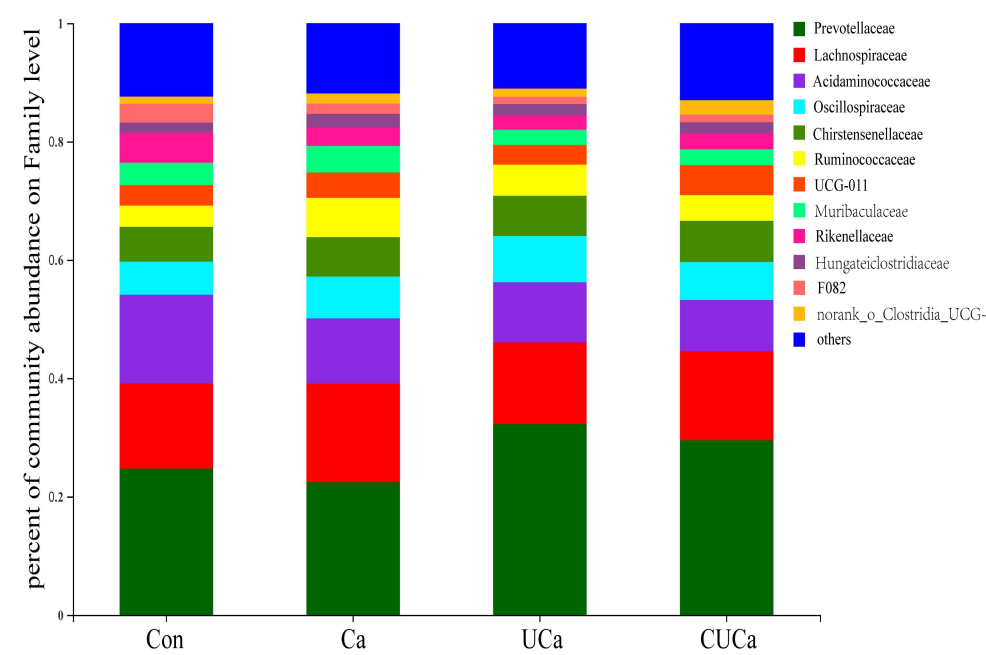

D

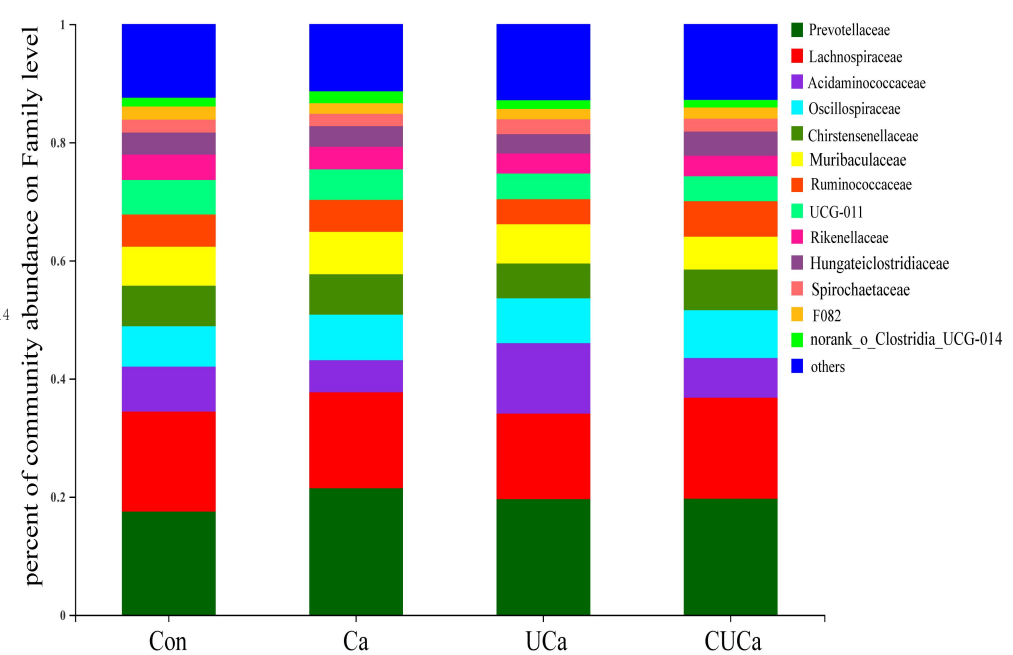

Supplement: Supplementary file 5 [file Image_3.pdf]

Figure S4

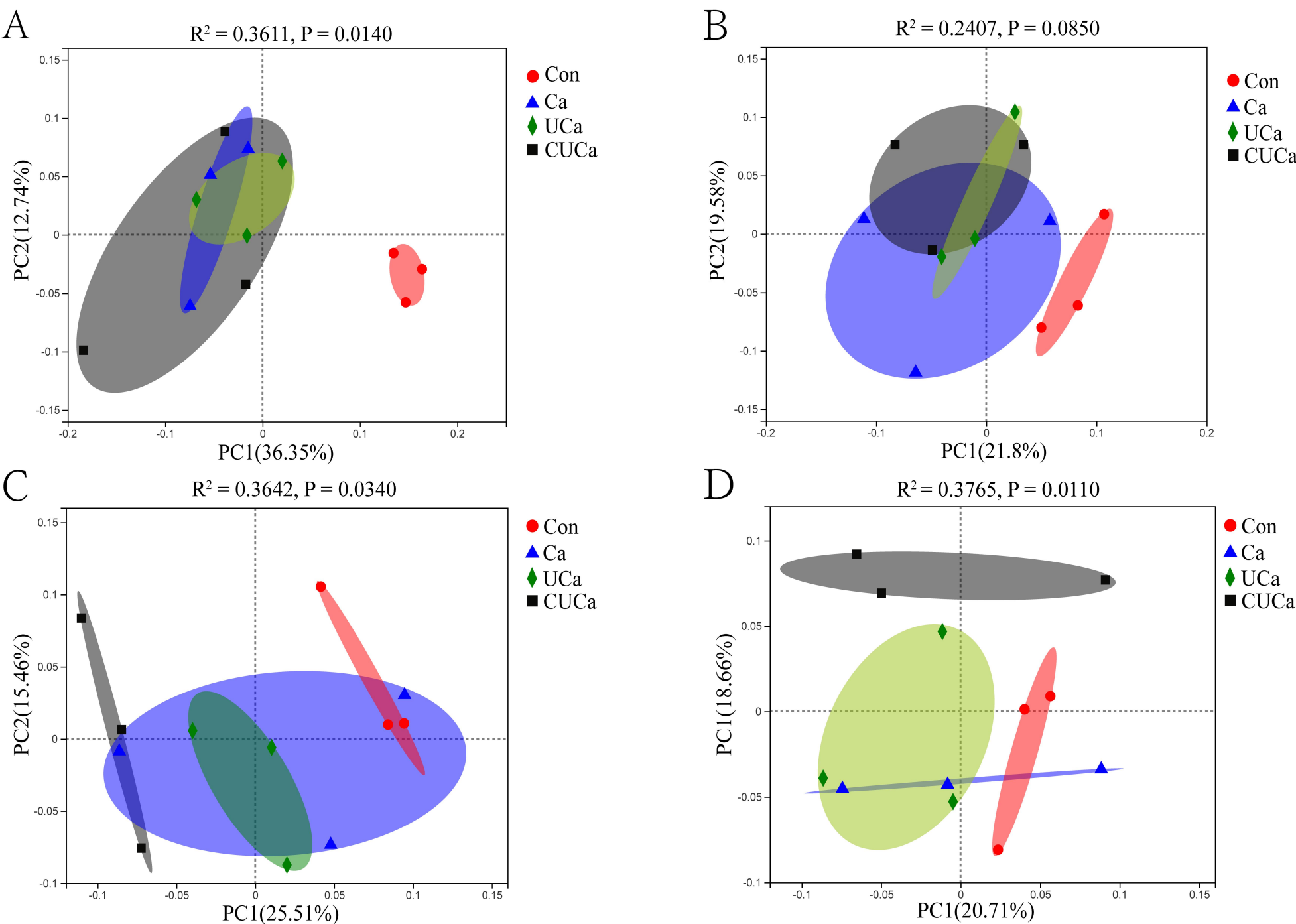

Supplement: Supplementary file 6 [file Image_4.pdf]

Figure S5

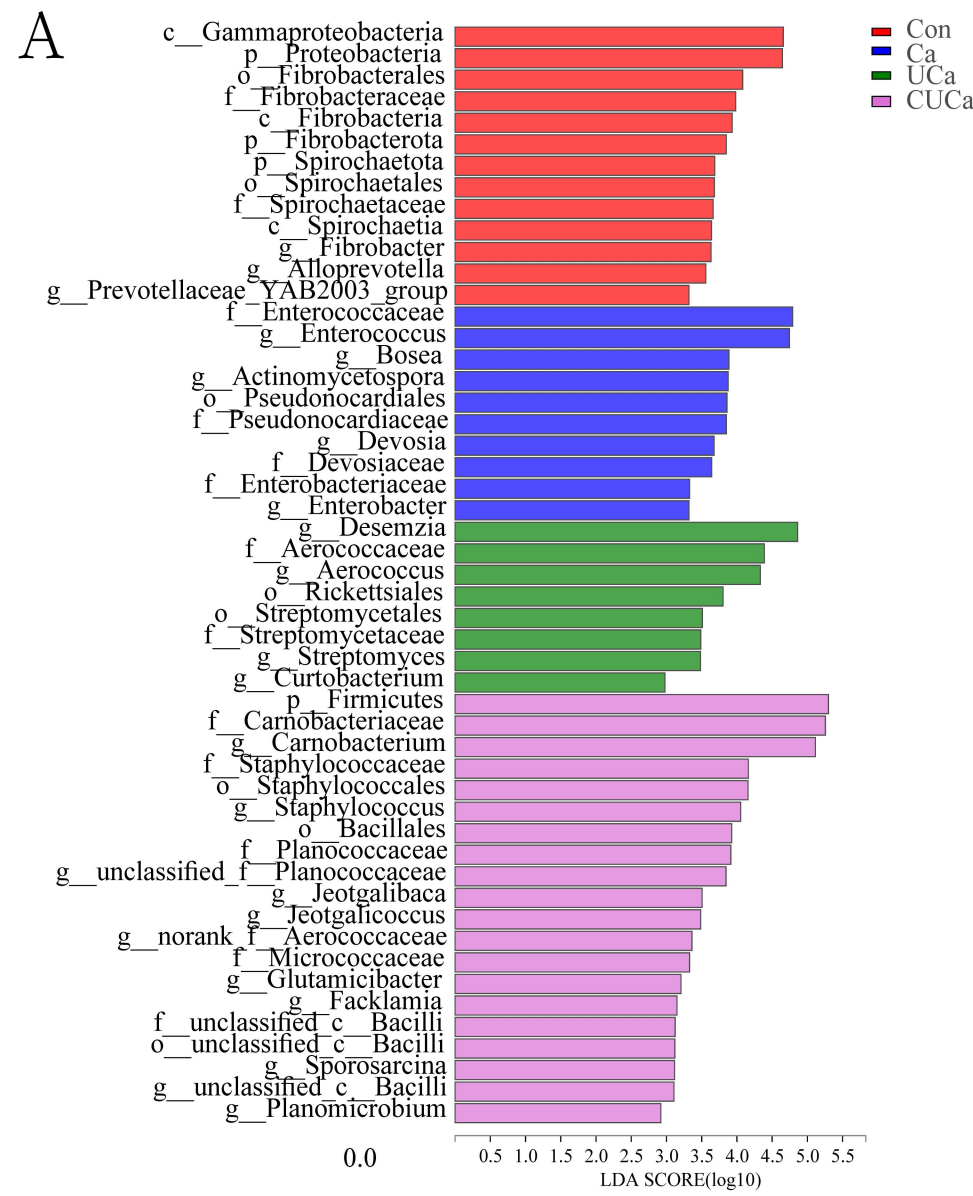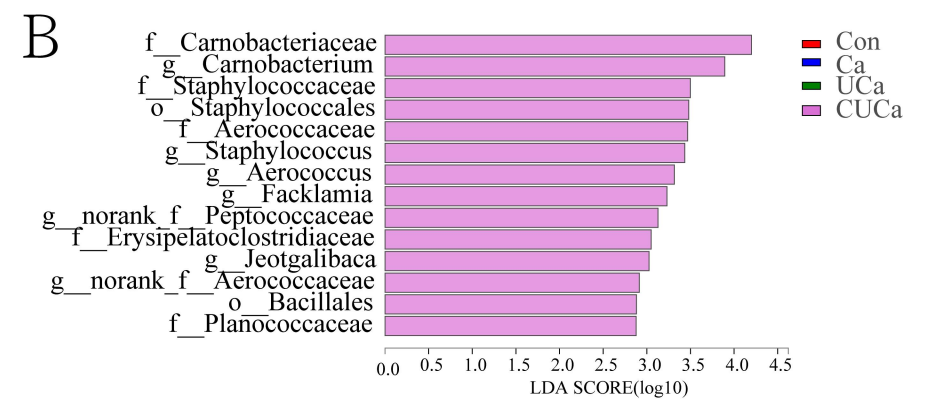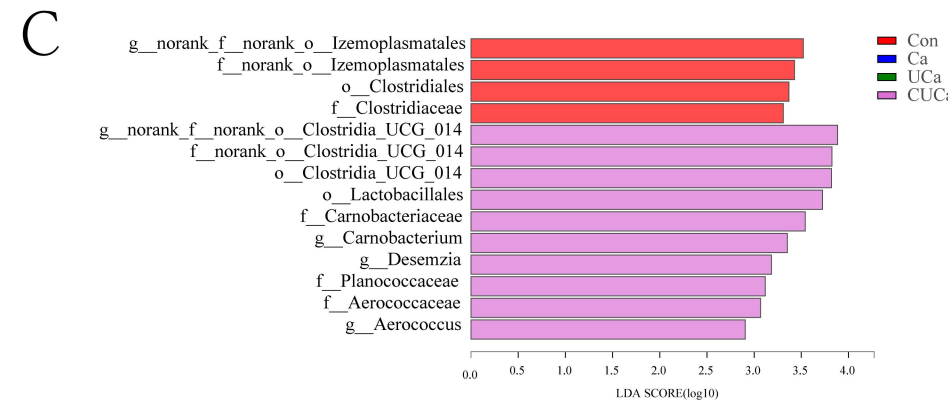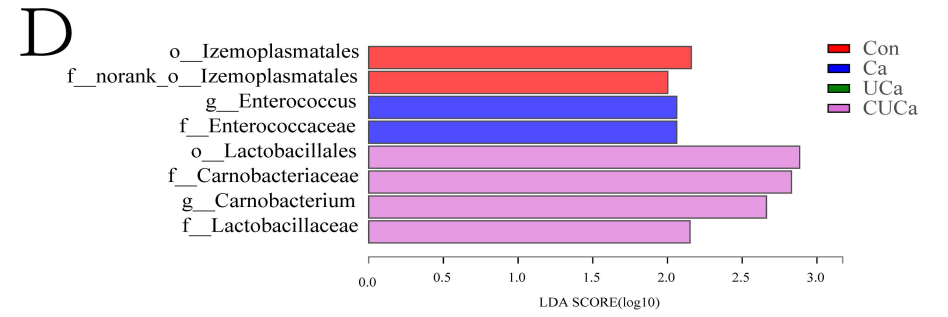

Supplement: Supplementary file 7 [file Image_5.pdf]
